# Editorial: What Is the Role for Effective Pedagogy in Contemporary Higher Education?

**DOI:** 10.3389/fpsyg.2018.01299

**Published:** 2018-07-30

**Authors:** Carl Senior, Dilly Fung, Christopher Howard, Rowena Senior

**Affiliations:** ^1^School of Life & Health Sciences, Aston University, Birmingham, United Kingdom; ^2^University of Gibraltar, Europa Point, Gibraltar; ^3^London School of Economics and Political Science, London, United Kingdom; ^4^Department of Psychology, Derby University, Derby, United Kingdom; ^5^Centre for Learning and Professional Practice, Aston University, Birmingham, United Kingdom

**Keywords:** higher education institutions, policy making, student satisfaction, consumer satisfaction, market access

“Improvise, Adapt and Overcome!”

Clint Eastwood, Heartbreak Ridge

Across the globe the Higher Education (HE) sector is undergoing a startling metamorphosis. No longer is HE the sole preserve of the privileged few; it is now for the masses. However, a new narrative is forming and it is one that clearly demarcates the role of the university and the student-here the student is the *consumer* of a product and not just a learner^1^. Students are now positioned as “entrepreneurs of the self” where HE is a “choice” to increase human capital and hence an individual's competitiveness within global economic markets (Foucault, 2008). Yet how far does a university have to go to embrace this consumer-centric narrative? There is a strong and respected body of evidence showing that a positive service encounter can indeed lead to a vast array of advantageous aspects such as customer loyalty, repeat patronage intentions and even positive word-of-mouth (e.g., Pugh, 2001; Caruana, 2002; Guenzi and Pelloni, 2004). Clearly these outcomes would be of great benefit to most, if not all, educational institutes. However, the very same body of literature also describes the need for customers to identify themselves within an authentic relationship (Tzokas et al., 2001). In light of the fact that the relationship between a student (customer) and University (service provider) is one that is sensitive to a variety of different outcomes that may be outside the control of the university administrators, such as postgraduate employability success and even (quite controversially) assessment success^2^ it is safe to say that there are a myriad of factors that may impact the vital service provider relationship between students and higher education institutions. Therefore, it may not be effective (or even common sense) to adopt a full consumer model just yet.

But consumer expectations *are* indeed central to a positive service encounter so an ambiguous attitude toward the relationship that the student and their University enjoys is likely to lead to anything but a positive experience (Goldney, 2008; Pinar et al., 2011). Now, is the time for institutional leaders to take a stand and declare the role that their students take in their learning and what position this plays in the larger organizational culture. To rephrase this stance within the narrative on consumer psychology one could ask, how does the student body actually inform the university brand such that the organization can develop an authentic relationship with its core customer base?

The ready embrace of consumerist ideology across the global HE sector will most likely see a rise of an open market structure that is highly sensitive to market forces (e.g., Porter, 2008). Economic theory (e.g., Fama, 1970) defines such a market place as one consisting of a large number of rational profit maximizers (e.g., universities) that try to predict future market values and where important information is freely available to all participants (e.g., the now central position of published student satisfaction metrics). One could quite easily argue that contemporary HE is firmly embedded within such an environment. Indeed, given the almost pathological obsession that some institutional managers have in spending money on a variety of student facing initiatives one can also be forgiven for thinking that we have embraced a form of “conspicuous consumption” that institutes are using to try and better their position in the global HE marketplace (Hamilton and Tilman, 1983; O'Cass and McEwen, 2004).

Yet while there are strong moves toward a more market-oriented consumer approach within HE a values-based resistance is forming. The papers that were submitted to this Research Topic are testament to the role of students not as consumers of a product, but as junior scholars, learners and co-creators of the experience at the very heart of effective pedagogy.

The papers included within this research topic can be generally divided into three sections with each relating to one aspect of effective practice in contemporary HE. The first of these sections focus on the expectations and practice of lecturing staff; Hassel and Ridout and Correia and Navarrete examine the differences in expectations and attitudes toward HE in both the student and teacher's mindset. Both identify the potential impact that a misalignment between the expectations of staff and students may have. Additionally, both make recommendations to ensure that teaching practice is aligned so it meets the expectations of the modern-day student. Cui et al. and Zhao and Zhang focus on the means by which increasing a teacher's enthusiasm can lead to an increase in professional identity, which ultimately leads to an improvement in the students' experiences. Bashir et al. demonstrate that students who enter HE via different routes demonstrate different levels of IT competency. This is an important finding as such competency often forms the bedrock of the transferable and professional skillset that, as Senior et al. found, the modern-day student seeks to obtain in HE.

The next set of papers delve deeper and uncover the mechanistic principles by which university practices can be aligned to meet student expectations. Senior et al. describe the very real need for universities to bring students to the very heart of its activity as true partners before it can deliver an effective pedagogy in these consumer-driven times. By adopting a student-as-partner narrative, it is possible to embed the lived experiences of students alongside the effective delivery of academic programmes (see e.g., Senior et al., 2014). As is seen with the work of Moores et al., compelling evidence supports the role of experiential work-based learning and the benefit that it has in supporting a more overarching and inclusive benefit. This theme is continued with Nash and Winstone, who consider the very core of the relationship between students and their university and examine how feedback is both delivered and received.

In the concluding collection of articles, Tissington and Senior and Knight and Senior both highlight institutional strategies that could be adopted to benefit the student learning experience. Finally, Sitaraman reminds us that we should not stray too far from our core purpose and that is to teach despite the various pressures that may result in a competitive marketplace.

In summary we provide three points to assist in getting the maximum benefit within this manifesto for effective practice:

- Embrace students as partners in all aspects of academic culture. Do not pay lip service to this relationship but instead develop real opportunities for students to engage. This is the authentic relationship that will lead to a positive student encounter.
- Drive only innovation that has proven to be effective. Do not succumb to the need for conspicuous consumption. The contemporary University should deliver excellence by meeting students' developmental needs. And finally,
- Do not believe the hype. A university can still deliver effective education even in times of obsessive consumerism.

## Author contributions

All authors listed have made a substantial, direct and intellectual contribution to the work, and approved it for publication.

### Conflict of interest statement

The authors declare that the research was conducted in the absence of any commercial or financial relationships that could be construed as a potential conflict of interest.

## Footnotes

^1^In the UK this consumer-based approach owes its birth to the publication of a series of influential government sponsored papers on the future of the UKHE sector that were published in 1999. These papers were collectively called “*The Reports of the National Committee of Inquiry into Higher Education*” but colloquially known as the “Dearing Report” after the lead author, Lord Ronald Dearing and it clearly initiated the movement that saw effective pedagogy move away from the traditional didactic arena and toward a more market structured environment.

^2^A good university will provide excellence in teaching to inspire effective learning that is assessed independently.
